# The Relationship Between Vitamin D and Postpartum Depression in Reproductive-Aged Iranian Women

**DOI:** 10.25122/jml-2018-0038

**Published:** 2018

**Authors:** Parvin Abedi, Maryam Bovayri, Ahmad Fakhri, Shayesteh Jahanfar

**Affiliations:** 1. Midwifery Department, Reproductive Health Promotion Research Center, Ahvaz Jundishapur University of Medical Sciences, Ahvaz, Iran; 2. Midwifery Department, Menopause Andropause Research Center, Ahvaz Jundishapur University of Medical Sciences, Ahvaz, Iran; 3. Psychiatry Department, Reproductive Health Promotion Research Center, Ahvaz Jundishapur University of Medical Sciences, Ahvaz, Iran; 4. School of Health Sciences, Health Professions 2239, Central Michigan University, Michigan, United States of America

**Keywords:** Vitamin D, postpartum depression, reproductive age, vitamin D deficiency, insufficiency

## Abstract

**Background:** The aim of this study was to evaluate the relationship between vitamin D and postpartum depression in reproductive-aged Iranian women.

**Methods and Results:** This study was conducted on 120 women (60 with postpartum depression and 60 without) in Izeh, Iran. A socio-demographic questionnaire and Beck Depression Scale were used for data collection. The ELISA method was used for measuring 25-OH vitamin D (ng). The participants were classified according to their vitamin D level as follows: 25-OH-D < 10ng/ml considered as severe deficiency, 10–20n g/ml as moderate insufficiency, 20–30 ng/ml as mild insufficiency and >30ng/ml as normal. Data were analyzed using the independent t-test or Mann-Whitney test, chi-square and logistic regression test. The mean level of vitamin D of women with postpartum depression was lower than that in normal women (16.89±7.05 vs. 21.28±7.13, p=0.001). More than 53% of women with postpartum depression had vitamin D <20 ng/ml compared to 31.7% of women with no depression (p=0.005). Moreover, 16.7% of women with postpartum depression had vitamin D < 10ng/ml compared to only 6.7% in the normal group (p = 0.005). Women with vitamin D less than 20ng/ml compared to vitamin D > 20ng/ml were 3.30 times more likely to have postpartum depression (OR: 3.3, CI: 1.32–8.24, p= 0.01).

**Discussion:** There is a significant relationship between a low level of vitamin D and postpartum depression among reproductive-aged Iranian women. Health policy makers should pay attention to the measuring vitamin D level as one of the primary tests of pregnant women.

## Introduction

Postpartum depression is a disorder that may be accompanied by the following conditions: insomnia, psychomotor changing in appetite, fatigue, feeling of guilt, worthlessness, and having suicidal thoughts that remain at least for two weeks postpartum [1]. According to the World Health Organization (WHO), globally 13% of women experience postpartum depression and this number reaches 19.8% in developing countries [2]. A systematic review of 41 studies showed that 25.3% of women in the postpartum period had depression [3]. Factors such as having a history of depression and anxiety during pregnancy, stressful life events, and low social support are considered important risk factors for postpartum depression [4]. Moreover, a review study by Mehta et al. showed that factors such as multiparity, unwanted pregnancy, premarital pregnancy, premenstrual syndrome, and history of postpartum depression are other significant risk factors for postpartum depression in Asian women [5].

Some physiological factors such as loss of proper functioning of the innate immune system and hypothalamic-pituitary-adrenal axis are proposed by Bodnar & Wisner in 2005 as a cause for postpartum depression [6]. Decreased levels of micronutrients such as n-3 PUFA, B vitamins, vitamin D and trace minerals are predisposing factors for postpartum depression [7]. A study on 687 pregnant women showed that pregnant women who had low levels of 25-OH vitamin D were more prone to have postpartum depression [8]. Furthermore, another study showed that women who had a low-level vitamin D in early pregnancy were at a greater risk for depression in mid and late pregnancy [9]. In contrast, Nielson et al., [10] in a case-control study on 605 women with postpartum depression and 875 controls found that there was no significant relationship between vitamin D and postpartum depression, but a high serum level of vitamin D was associated with postpartum depression. Another study also confirmed an association between a higher level of inflammatory markers and low levels of prenatal vitamin D and postpartum depression [11]. Although Vaziri et al. showed that consumption of 2000 IU of vitamin D could significantly reduce the depression score at 38–40 weeks of gestation and also after birth [12], there is still a paucity of information regarding vitamin D level among Iranian pregnant women and postpartum depression. The vitamin D levels of Iranian women are lower than those in other countries. According to recent research in Iran, the prevalence of vitamin D deficiency was 69% and 20.3% of women aged 20–80 years had a severe vitamin D deficiency [13]. Therefore, the primary aim of this study is assessing the relationship between vitamin D and postpartum depression in reproductive-aged Iranian women.

## Material and Methods

This was a case-control study in which 60 women with and 60 women without postpartum depression were recruited in Izeh, Iran. Izeh is one of the cities of the Khuzestan province that is located in the Northeast of the province and has a latitude of 31° 50’ 2.7” (31.8341°) North. This study started in November 2016 and was completed in May 2017. This time period involves winter and spring in this region.

The design of this study was approved by the Ethics Committee of Ahvaz Jundishapur University of Medical Sciences, Ahvaz, Iran (Ref No: IR. AJUMS.REC.1395.463) in accordance with the ethical standards laid down in the 1964 Declaration of Helsinki and its later amendments. All participants gave informed consent before data collection. The inclusion criteria were as follows: women aged 18–35 years and at 6–8 weeks after childbirth. Women who had a neonate admitted to the Neonatal Intensive Care Unit (NICU), and those with a history of mental disorders, who had a newborn with congenital anomalies, repeated caesarian section, gestational diabetes, preeclampsia [14] and thyroid disorders [15] during pregnancy were excluded from the study. The sample size was calculated according to a pilot study on 20 women in their postpartum period as follows:

**Figure:**
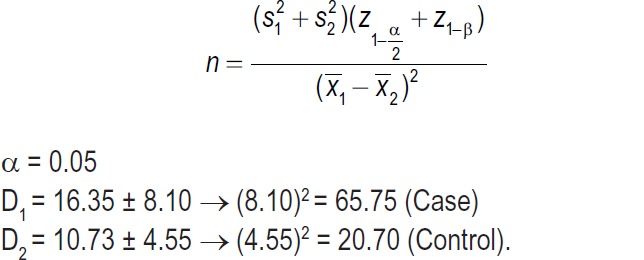


Eligible women were selected from public health centers in Izeh, Iran. All women in the case and control groups were matched regarding age and taking vitamin D supplements.

## Outcome Measures

In this study, a socio-demographic questionnaire and the Beck Depression Scale [16] were used for data collection. The Beck scale included 21 questions with 4 answers, scoring from 0 (good condition without any depression) to 3 (the worst condition). The total score of this scale was between zero and 63. Scores between 1–10 indicate normal condition, 11–16 show mild mood disturbance, 17–20 indicate moderate depression, 31–40 indicate severe depression and > 40 indicate extreme depression. The validity and reliability of the Beck Depression Inventory were approved by Ghassemzadeh et al., in Iran [17]. The demographic questionnaire and the Beck Depression Scale were completed through interviews.

For measuring vitamin 25-dihydroxy vitamin D (25-OH-D) level, 5mL venous blood was drawn from each participant and sent to a reference laboratory for centrifuge (Izeh, Iran). The separated sera were kept in –30 °C, until all samples were completed, and then, while being kept in a cold box, were transferred to a reference laboratory in Ahvaz (Iran Zamin) for measuring 25-OH vitamin D. The ELIZA method from immunodiagnostic System Limited validated against HLPC method was used to measure 25-OH vitamin D (Monobind Company, USA) and all measurements were reported by nanogram. Participants were classified into the following groups based on the amount of vitamin D: 25-OH-D<10ng/ml and 10–20ng/ml were considered as severe deficiency and moderate insufficiency respectively, 20–30ng/ml as mild insufficiency and >30ng/ml was considered normal. The level of vitamin D (25 OH D) has a biological half-life of three weeks, makes the measuring of this element the most reliable indicator of vitamin D [18].

### Statistical Analyses

All data were entered into SPSS version 22. The normality of continuous data was checked using the Kolmogorov–Smirnov test. For comparing continuous data, either the independent t-test or the Mann-Whitney test was used. The chi-square test was used for comparing categorical data. The logistic regression analysis was conducted for assessing the relationship between vitamin D and depression adjusting for age, education, education of husband, economic situation and body mass index. A p-value <0.05 was considered significant.

## Results

Table 1 shows the socio-demographic characteristics of participants with depression and those of the normal group. As evident from this table, the body mass index (BMI) in women with postpartum depression was higher than that in normal women; however, this difference was not significant. Women with postpartum depression had less sunlight exposure than women with no depression (9 ± 13.01min/week vs. 10.53 ± 13.86 min/week, p = 0.48). The two groups did not have any significant difference regarding education (p= 0.63), education of husband (p=0.62) and occupation (p=0.69). The percentages of women with a good socio-economic situation were significantly higher in women without postpartum depression than that in women with postpartum depression (23.3% vs. 6.7%, p=0.01).

The two groups also did not show any significant difference regarding mode of delivery, breastfeeding during the 6 weeks after delivery, history of abortion and the number of pregnancies. Women in the postpartum depression group had significantly more undesired pregnancies than those in the normal group (p=0.002). (Table 2).

**Table 1:** Sociodemographic characteristics of women with and without postpartum depression

Variables	Women with postpartum depression n=60	Women without postpartum depression n=60	P value
**Mean (SD)**			
Age (y)	26.43±4.27	27.6±4.73	0.16
Body mass index (kg/m^2^)	24.89±3.5	25.33±3.33	0.48
Sunlight exposure (min/day)	9±13.01	10.53±13.86	0.48
**N (%)**			
**Education**			
Primary	5(8.3)	5(8.4)	0.63
High school	14(23.3)	10(16.7)	
Secondary high school	31(51.7)	37(61.7)	
University education	10(16.7)	8(13.3)	
**Education of husband**			
Primary	8(13.3)	4(6.7)	0.62
High school	17(28.3)	16(26.7)	
Secondary high school	32(53.3)	36(60)	
University education	3(5)	4(6.7)	
**Job**			
Home maker	56(93.3)	57(95)	0.69
Employee	4(6.7)	3(5)	
**Economic situation**			
Good	4(6.7)	14(23.3)	0.01
Moderate	48(80)	43(71.7)	
Weak	8(13.3)	3(5)	

**Table 2:** Midwifery characteristics of participants in two groups: with and without postpartum depression

Variables	Women with postpartum depression n=60	Women without postpartum depression n=60	P value
**N (%)**			
**Mode of delivery**			
Normal vaginal delivery	43(71.7)	40(66.7)	0.55
Cesarean section	17(28.3)	20(33.3)	
**Pregnancy**			
Desired	45(75)	57(95)	0.002
Undesired	15(25)	3(5)	
**Breastfeeding during 6 weeks after delivery**			
Yes	58(96.7)	59(98.3)	0.559
No	2(3.3)	1(1.7)	
**History of abortion**			
Yes	5(8.3)	2(3.3)	0.439
No	55(91.7)	58(96.7)	
**Number of pregnancy**			
First	44(73.3)	41(68.3)	0.201
Second	13(21.7)	9(15)	
Third	2(3.3)	6(10)	
Forth	1(1.7)	4(6.7)	

**Table 3:** Level of serum vitamin D in two groups with and without postpartum depression

Variables	Women with postpartum depression n=60	Women without postpartum depression n=60	P value
**Mean ±SD or N (%)**			
Mean of vitamin D (ng/mL)	16.89±7.05	21.28±7.13	0.001
Vitamin D level (ng/mL)			
>30	2(3.3)	7(11.7)	0.005
21-29	16(26.7)	30(50)	
<20	32(53.3)	19(31.7)	
<10	10(16.7)	4(6.7)	

Table 3 shows the level of vitamin D in women with postpartum depression and that of the non-depressed women. As evident from this table, the mean of vitamin D was significantly lower in women with postpartum depression compared to normal women (16.89±7.05ng/ml vs. 21.28±7.13ng/ml, p=0.001). The number of women with vitamin D<20ng/ml was significantly higher in the postpartum depression group compared to the normal group (53.3% vs. 31.7%, p=0.005). Also, 16.7% of women with postpartum depression had vitamin D<10ng/ml compared to only 6.7% in the normal group (p=0.005).

The relationship between postpartum depression and some variables was assessed using multivariate logistic regression. As evident from Table 4, women with vitamin D<20ng/ml compared to vitamin D>20ng/ml were 3.30 times more likely to have postpartum depression (OR: 3.3, CI: 1.32–8.24, p= 0.01). There was also a significant relationship between age and postpartum depression (OR: 1.14, CI: 1.02–1.27, p=0.01). Women with a good economic situation were 7.48 less likely to have postpartum depression (OR: 7.48, CI: 0.96–57.7, p= 0.054). Women with desired pregnancies were 4.80 times less likely to have postpartum depression (OR: 4.80, CI: 1.11–20.61, P=0.02). There was no relationship between education, education of husband and job with postpartum depression in the adjusted model.

## Discussion

This study was designed to test the relationship between vitamin D and postpartum depression in reproductive-aged Iranian women and vitamin D deficiency is more prevalent in Iranian women.

**Table 4:** The relation of some variables with postpartum depression using logistic regression

Variables	OR crude (95% confidence interval)	OR adjusted (95% confidence interval)	P value
Vitamin D <20ng/ml compared to >20ng/ml	4.33(1.83-10.24)	3.30 (1.32-8.24)	0.01
Age	1.10(0.99-1.21)	1.14 (1.02-1.27)	0.01
Body mass index (kg/m^2^)	1.001(0.90-1.15)	0.98 (0.86-1.13)	0.87
Primary education	1.37(0.15-11.85)	1.88(0.18-19.1)	0.59
High school	0.91(0.16-5.10)	1.27(0.19-8.59)	0.80
Secondary high school	1.10(0.28-4.26)	2.16(0.46-10.02)	0.32
Husband education (Primary)	0.17(0.01-2.052)	0.09(0.006-1.30)	0.07
Husband education (high school)	0.43(0.05-3.33)	0.32(0.03-2.71)	0.29
Husband education (secondary high school)	0.73(0.11-4.9)	0.40(0.05-2.99)	0.37
Good economic situation	0.38(0.05-2.90)	7.48(0.96-57.7)	0.054
Moderate economic situation	0.33(0.57-1.94)	0.91(0.18-4.65)	0.91
Home makers	3.17(0.45-22.2)	2.49(0.35-17.71)	0.36
Desired pregnancy	4.76(1.18-19.18)	4.80(1.11-20.61)	0.02

Reference for education: University education Reference for Economic situation: Weak Reference for job: Employees Reference for pregnancy: Undesired pregnancy

A study by Kazemi et al. on 67 women who gave birth vaginally showed that the mean maternal serum of 25-OH-D was 19.4 ± 3.9 nmol/l, cord blood 25 (OH) D was 16.7 ± 2.9 nmol/l, and 86% of mothers and 75% of newborns had low levels of vitamin D [19].

Also, a systematic review of 48 studies with 18,531 participants conducted in Iran showed that the prevalence of vitamin D deficiency was 45.64%, 61.9% and 60.45% among male, female and pregnant Iranian women respectively [20].

A study by Maghbooli et al. on 552 pregnant women in Tehran (the capital of Iran) showed that the prevalence of vitamin D deficiency in mothers and their neonates was 66.8% and 93.3% respectively, and there was a significant relationship between maternal and neonatal vitamin D deficiency [21]. For this reason, the Iranian Ministry of Health and Medical Education distribute mineral supplements (containing 400 IU vitamin D) among pregnant women in all public health centers [22].

Also, the prevalence of postpartum depression in Iranian women is increased compared to other countries. A systematic review by Veisani et al. showed that the prevalence of postpartum depression was 25.3% (95% CI: 22.7–27.9), and undesired pregnancy, being a housewife, having a history of postpartum depression significantly contributed to postpartum depression [3]. The prevalence of postpartum depression in developed countries is 10–20% [23].

Studies have shown that 1,25 OH-D [3] as a hormone is a modulator of the immune system and receptors of this vitamin are located in resting and activated lymphocytes (mostly in CD8 and CD4 lymphocytes) [24]. One possible mechanism for causing depression in the case of vitamin D deficiency is the increased concentration of Ca^2+^ inside the inhibitory neurons caused by N-methyl-D-aspartate (NMDA) receptors. Vitamin D can reduce the level of Ca^2+^ and thereby decrease depression [25].

### Findings of the Present Study

Our results showed that women with postpartum depression had a lower mean of 25-OH-D. Also, the number of women with moderate insufficiency and severe deficiency was significantly higher in the postpartum depression group compared to normal women.

Contrary to our findings, Nielsen et al., who examined the level of 25 OH-D3 during pregnancy and its relationship with postpartum depression among 605 women with postpartum depression and 875 controls, found that there was no relationship between the concentration of vitamin D and postpartum depression [10]. The reason for this discrepancy may be the fact that Nielsen et al. collected blood samples in the 24^th^–25^th^ weeks of gestation while we measured vitamin D concentration 6–8 weeks after giving birth.

A study by Vaziri et al. showed that the prenatal consumption of vitamin D supplements (2000 IU) starting from the 26^th^ - 28^th^ week of gestation could significantly reduce the depression score in the vitamin D group compared to placebo in 38^th^ – 40^th^ weeks of gestation and 4–8 weeks after childbearing (p<0.05) [12]. These results are in line with ours.

Our results showed that women with vitamin D less than 20ng/ml compared to vitamin D>20ng/ml were 3.30 times more likely to have postpartum depression. Huang et al. recruited 498 women in their early gestation for a study and assessed the relation between vitamin D and depression and anxiety. Results showed that with a 1ng/mL decrease in 25[OH] D, the risk of anxiety and depression increased by 0.043 and 0.040 (p-values=0.052 and 0.029, respectively) [26]. The results of Huang et al. are in line with our study.

Our results also showed that undesired pregnancy, age and poor economic situation are risk factors for postpartum depression. Other studies also showed that there is a relationship between postpartum depression and low socioeconomic status [27] and unplanned pregnancy [28].

### Strengths and Limitations of the Study

The numbers of studies that have addressed the relationship between vitamin D and postpartum depression is very small, and to the best of our knowledge, no study has ever been conducted among Iranian women. Although Vaziri et al. conducted a study to evaluate the effect of vitamin D3 supplementation (2000 IU/day) during pregnancy on perinatal and postpartum depression score, further studies on this topic are still warranted [12]. Our study may be limited as collected data about taking supplements and sunlight exposure may be affected by recall bias. Also, data collection and vitamin D measurements have been done in the two seasons (winter and spring) which may affect the level of vitamin D.

The results of this study showed that the level of serum vitamin D was significantly lower in women with postpartum depression, and those with severe deficiency of vitamin D were twice more likely to have postpartum depression. Due to the type of women’s clothing in Iran, the absorption of vitamin D through the skin is very low. Therefore, health policymakers should pay attention to the fact that measuring the vitamin D level should be considered one of the primary tests for pregnant women so that they could be treated accordingly.

## Acknowledgment

This study was a master thesis of MB. All expenses of this study were provided by Ahvaz Jundisahpur University of Medical Sciences, Ahvaz, Iran. We would like to thank all the women who participated in this study.

## Conflict of Interest

The authors confirm that there are no conflicts of interest.
